# Peeling Affects the Nutritional Properties of Carrot Genotypes

**DOI:** 10.3390/foods11010045

**Published:** 2021-12-24

**Authors:** Giulia Conversa, Anna Bonasia, Giuseppe Natrella, Corrado Lazzizera, Antonio Elia

**Affiliations:** 1 Department of Agriculture, Food, Natural Resources and Engineering (DAFNE), University of Foggia, 71100 Foggia, Italy; anna.bonasia@unifg.it (A.B.); corrado.lazzizera@unifg.it (C.L.); antonio.elia@unifg.it (A.E.); 2 Department of Soil, Plant and Food Sciences (DiSSPA), University of Bari Aldo Moro, Via G. Amendola, 165/A, 70126 Bari, Italy; giuseppe.natrella@uniba.it

**Keywords:** local varieties, root flesh, carotenoids, phenols

## Abstract

Peeling may result in changes in carrot’s nutritional properties; therefore, the present study focused on its effect on the retention of principal nutrients (minerals, sugars, organic acids) and antioxidants (carotenoids and phenols) in the peeled roots of two landraces (‘Carota a punta lunga’—CPL and ‘Carota a punta tonda’—CPT) and a hybrid cultivar (‘Presto’) grown in the area of the “Salterns of Margherita di Savoia” area (Puglia region). The peel had a higher concentration of cations (+92%), organic acids (+103%), carotenoids (+42%), and phenolic acids (seven times) than root flesh. For each chemical class, the most abundant components were K, malic acid, ß-carotene, and chlorogenic acid, respectively. The two landraces stand out for the accumulation of the phenolic acids and ß-carotene, whereas the peel of ‘Presto’ was distinguished by the concentration of Ca and ascorbic and pyruvic acids. The root flesh had a greater accumulation of simple sugars, nitrate (mainly in CPL), oxalic acid, and in particular in the flesh of ‘Presto’, of Na and Cl. For local varieties, peel removal seems to impact the nutritional and antioxidative properties of carrots more consistently compared to the advanced cultivar, since it represents on average 21% and 59% of the total carotenoids and phenols, respectively, of the intact roots.

## 1. Introduction

The carrot (*Daucus carota* L.) is among the most important root vegetables cultivated worldwide. According to the United Nations Food and Agriculture Organization (FAO), the world production of carrots and turnips (these plants are combined by the FAO) was about 45 million tonnes in 2019, with China ranking first (47.9% of the world total production), followed by Uzbekistan (6.2%), the U.S.A (5.0%), the Russian Federation (3.5%), Ukraine (1.9%), the U.K. (1.8%), and Germany (1.8%) as other main producers. Italy, with about 11,000 ha grown annually, ranks 17th [1].

The most common carrots are the orange-coloured type; however, yellow or purple-coloured roots are also produced in Europe, the U.S.A., Turkey, and India [2].

This vegetable has gained in popularity due to increased awareness of its nutritional value [3], since it is rich in nutrients and health-promoting compounds, including minerals, sugars, carotenoids, and phenols [4]. Recent meta-analysis studies, considering the relationship between carrot consumption and the development of some human diseases, have highlighted the key role of this vegetable in contrasting breast, gastric, lung, and prostate cancer [5,6,7,8]. These proprieties could be associated with its high content in carotenoids and phenolic compounds able to scavenge free radicals to improve the defence system against oxidative stress [9].

Carrots are consumed either as processed (meals and juices) or fresh roots; specifically, the consumption of fresh-cut carrots is increasing due to changes in consumer behaviour [10]. Both processed and fresh carrots handling involves the production of wastes, including peel, which may represent a good source of important bioactive compounds. However, removing the peels may result in changes in the nutritional/antioxidant properties of peeled products. In fresh and unstored carrots, a reduction in phenol compounds [10,11,12] as well as in carotenoid content [12] has been observed when peeled. Similarly, puree obtained from unpeeled carrots contained higher levels of phenols, carotenoids, and sugars compared with manually peeled roots [13]. Concerning phenols, despite peel scarcely contributing to the total root fresh weight, it is reported to contain more than 50% of the total of these compounds [9]. Moreover, it has been highlighted that solutes such as simple sugars (fructose, glucose, sucrose), inorganic ions, and organic acids, affecting both nutritional and organoleptic (taste, flavour) characteristics [14], are differently accumulated in root tissues, with the periderm being richer in ions, in an opposite trend to sugars [15].

Both pre-harvest (climate, cultivation site and system, genotype) and post-harvest factors (storage conditions, processing typology) affecting biosynthesis and/or the accumulation of such nutrients are well thorough out [9]. However, to the best of our knowledge, there are very few studies focused on nutrients and antioxidants distribution in peels and the inner part (cortex and core) of carrot root underlining differences between genotypes [11].

In the Puglia region (Southeast Italy), the cultivation of carrot is spread over 1,110 ha, which is mostly concentrated in the provinces of Foggia (650 ha) and Barletta–Andria-Trani (BT) (200 ha) area, and it represents 10% of the total national cultivation [16]. It is mostly based on hybrid commercial genotypes for fresh consumption; however, the cultivation of some local landraces persists in Puglia [17], supplying local retail markets (‘yellow-purple Polignano’ carrot, ‘Tiggiano’ carrot) [2,18,19,20].

The introduction of modern cultivars has strongly contributed to the loss of genetic agro-diversity, and vegetable landraces are considered highly susceptible to genetic erosion [21]. The potential of the exploitation of landraces is mostly associated with the high content of bioactive and antioxidant compounds such as phenols and carotenoids in carrot landraces [2,18,19,20], glucosinolates and vitamins in turnip landraces [22], and phenols in garlic landraces [23]. These compounds represent an added value that could push the preference of consumers towards landrace products.

Recent studies performed both on landraces and a commercial cultivar cultivated in the area of the “Salterns of Margherita di Savoia” area (FG and BT provinces) have pointed out the high nutritional profile of these genotypes compared with literature data, which is mainly due to peculiarities of the cultivation site (*arenili*) along with the genetic characteristics. In particular, these landraces stand out for their phenol and β-carotene contents [24].

In order to provide a comprehensive characterization of these carrots, the main objective of the present study was to evaluate the effect of peeling on the retention of principal nutrients (minerals, sugars, organic acids) and antioxidants (carotenoids and phenols) in the peeled roots.

## 2. Materials and Methods

### 2.1. Plant Materials, Collecting Site and Sampling

In March 2018, the same carrot genotypes considered in our previous work were collected from the same fields located in the “Salterns of Margherita di Savoia” area, around Margherita di Savoia (BT) village (41°22′31″ N; 16°9′13″ E) (0–10 m a.s.l.) (Figure S1) characterized by hot summers and mild winters with quite scarce rain, which is mostly concentrated in the late autumn–winter period [24]. Genotypes were two landraces named by the growers as “Carota a punta lunga” (CPL, meaning: elongated tip carrot) (Imperator type) and “Carota a punta tonda” (CPT, meaning: rounded tip carrot) (Nantes type) (Figure S2). Both have been developed from ancient cultivars introduced in this area, which then were selected and auto-propagated by farmers over the last 50 years. Currently, some farmers still cultivate these landraces in small areas, and they are also conserved at the genebank of the Institute of Biosciences and Bioresources (IBBR-CNR), Bari. 

The hybrid F1 Presto (orange carrot, Nantes type) (Vilmorin-Mikado, Fano, PU, Italy) was also collected, since it is largely cultivated in this area and other Puglia region areas.

The fields are located in the *arenili* which are sandy shores, laid out in a comb system of long narrow field strips suitable for the cultivation of root vegetables in the autumn–winter cycle. More details on the origin and characteristics of carrot landraces as well as a description of the *arenili* are reported in Bonasia et al. [24].

For each genotype, the fresh weight and number of leaves were measured on 15 plants, whereas samples of 20 ± 0.5 kg of carrots were mixed to obtain three independent replicates (50 roots each). They were rinsed in order to remove soil contamination; subsamples (10 roots for each replicate) were obtained to perform fresh weight, equatorial, and longitudinal diameter measurements in duplicate.

The remaining roots were topped, tailed, and manually peeled using a hand-held peeler to remove the periderm (approximately 2 mm). The fresh and dry weight was measured on both peeled roots and peel (in duplicate for each replicate); the chemical analyses were performed on a representative sample of the fresh peel and root flesh (in triplicate for each replicate) obtained as reported in Bonasia et al. [24]. The concentration of inorganic cations and anions, organic acids, simple sugars, phenolic compounds, lutein, and ß-carotene were all measured. Moreover, the sweetness index was determined.

The morphological measurements (root equatorial and longitudinal diameter) were performed using images from an image acquisition station [24]. The dry matter concentration was calculated as dry weight (dw)/fresh weight (fw)*100, whereas fresh material was freeze-dried in order to determine the dw.

Details about chemical determinations are reported in the previous work [24], following a brief description.

### 2.2. Standards and Reagents

Glucose, fructose, and sucrose standards, standards for phenolic (caffeic, chlorogenic, and di-caffeoyl-quinic acid) and all the other standards, HPLC-grade methanol, methanesulfonic acid, and acetonitrile, sodium hydroxide, sodium carbonate, and sodium bicarbonate were purchased as reported in Bonasia et al. [23]. Ultrapure water (18.2 MΩ) was produced through a Milli-Q water purification system (Millipore, Germany).

### 2.3. Inorganic Cations and Anions

Inorganic ions were analysed by ion chromatography (Dionex ICS 3000; Dionex-ThermoFisher Scientific, Waltham, MA, USA) equipped with an isocratic pump and conductivity detector as detailed in Bonasia et al. [23].

### 2.4. Simple Sugars and Sweetness Index

Simple carbohydrates were extracted from 30 mg of lyophilized samples treated according to Rohrer [25] and analyzed using an ICS 3000 System (Dionex-ThermoFisher Scientific, Waltham, MA, USA) by an anion-exchange column Carbopac PA1 column 4 × 250 mm combined with pulsed amperometric detection (ED50) (ThermoFisher Scientific, Waltham, MA, USA) maintained at 30 °C. Carbohydrates were identified by a comparison of the retention times with those of sugar standards. Peak areas were analyzed using Dionex Chromeleon software (version 6.80, Dionex-ThermoFisher Scientific, Waltham, MA, USA).

The sweetness index (SI) was assessed as reported in Magwaza and Opara [26].

### 2.5. Carotenoids

ß-carotene and lutein were extracted from lyophilized samples (0.1 g) and quantified as reported by Taungbodhitham et al. [27] with some modifications. Quantitative analyses of ß-carotene and lutein were carried out using a gradient HPLC method with an ICS 3000 System (Dionex-ThermoFisher Scientific, Waltham, MA, USA), as described in Bonasia et al. [24].

### 2.6. Organic Acids

Organic acids were extracted from the lyophilized sample (0.3 g) by using the Gonzalez–Castro et al. [28] method with some modifications. Organic acids were separated by an ICS 3000 HPLC System (Dionex-ThermoFisher Scientific, Waltham, MA, USA) equipped with an isocratic pump, Hydro-RP 80A column (250 × 4.60 mm) (Phenomenex Inc., Castel Maggiore, BO, Italy), which was maintained at 30 °C combined with a UV-visible detector (RLSC Diode Array Detector, 210 nm). Individual organic acids were identified by comparing retention times and UV-visible spectra with those of available standards. Peak areas were analyzed using Dionex Chromeleon software (version 6.80, Dionex-ThermoFisher Scientific).

### 2.7. Phenolic Compounds

Phenolic compounds were extracted from the lyophilized sample (0.05 g) with the methodology of Pasqualone et al. [29] with some modifications. The analysis was performed using the Ultra-High-Performance Liquid Chromatography (UHPLC) Dionex Ultimate 3000 RS system (Dionex-ThermoFisher Scientific, Waltham, MA, USA). The phenolic compound separation was achieved using a Hypersil GOLD aQ C18 column, 100 mm in length, 2.1 mm ID, and 1.9 μm particle size (Waters, Milford, MA, USA) maintained at 30 °C. Details on the mobile phase and Mass Spectrometry (MS) conditions are reported in Bonasia et al. [24]. Phenolic compounds were identified by comparing elution times, molecular ions, and MS/MS fragmentation patterns of the experimental spectra with those obtained from the available pure standard compounds or by tentative methods using reported data from the literature. Calibration curves were created to obtain quantification results and were based on the UV signal of each available standard. When no commercial standard was available, a similar compound from the same phenolic group was used as a standard.

### 2.8. Statistical Analysis

Data were processed through one-way ANOVA using Statistical Analysis Software (SAS, Cary, NC, USA). Mean separation was performed using the least significant difference (LSD) test (*p* = 0.05). Principal Component Analysis (PCA) was performed for visual analysis of data, using the PAST3 Software [30]. The data matrix considered all genotypes and root portions with relative replications. The data were standardized ((x-mean)/standard deviation) before the PCA analysis.

## 3. Results and Discussion

### 3.1. Bio-Morphological Traits

The local carrot genotype “Carota a punta lunga” (CPL) exhibited a higher leaf number and fresh weight along with larger roots (*p* ≤ 0.001) (greater fresh weight, length, and equatorial diameter) compared with the landrace “Carota a punta tonda” (CPT) and the commercial genotype ‘Presto’ (Table 1), pointing out its greater plant vigour. This is also confirmed by the observations reported by farmers who have managed these cultivations for decades.

Variability in dry matter (DM) concentration was observed between genotypes, with CPT and ‘Presto’ showing, respectively, the lowest (*p* ≤ 0.001) and the highest (*p* ≤ 0.001) values of both peeled carrots and peel. Moreover, for each genotype, the DM concentration of peel was lower than that of peeled roots (Table 1). The peel/total root fresh weight ratio was 11% for CPL and ‘Presto’, and it was 15% for CPT (*p* ≤ 0.001). Despite presenting a decreased DM in comparison to the same varieties grown in the same area, as reported in our previous study [24], an averaged high root DM level for the studied genotypes (10.5 g 100 g^−1^ fresh weigh –fw) was confirmed, suggesting that they have a prolonged shelf-life [31].

### 3.2. Inorganic Ions

The cv. Presto showed the highest concentration in both portions of Ca, Mg, and Na and the poorest in K (*p* ≤ 0.001), while the peel and root flesh of CPL roots were the highest in K concentration. In both portions, no differences emerged for Ca, Mg, and Na concentrations between landraces, except for a lower concentration of Ca detected in the peel of CPT compared to CPL (Table 2).

In both portions, the most abundant cation was K, which was followed by Na, Ca, and Mg, supporting findings reported by Nicolle et al. [32]. Averaged over genotypes, a greater level of total inorganic cations was accumulated in peel (+92% compared with peeled root) especially due to K concentration (Table 2). In agreement with our results, the concentration of this cation was observed to increase in the root periderm compared to the inner part by Korolev et al. [15]; however, information about other cations was not reported in this latter work.

It is noteworthy that from a human nutrition point of view, vegetables with a low Na/K ratio may prevent hypertension [33]. In this study, it emerged that for the cultivar Presto, the “Na/K” ratio always reached values higher than the landraces (Table 2). The tendency of this hybrid to accumulate less K and more Na than CPL and CPT had already been observed in the previous work [24] and also confirmed by Cefola et al. [18] comparing the same commercial genotype with the ‘yellow-purple Polignano carrot’ landrace. Additionally, our work highlights that due to the radial distribution of K, the highest Na/K ratio occurred in the peeled portion of the commercial genotype, whereas for the landraces, it was comparable to other works [18,24].

Variability emerged between genotypes for individual and total anion concentration with CPT having higher concentrations of Cl and lower SO_4_ both in root flesh and peel (*p* ≤ 0.001) along with lower PO_4_ concentration in flesh (*p* ≤ 0.001) (Table 3).

It is noteworthy that the CPL landrace stood out for the highest NO_3_ concentration (*p* ≤ 0.001), confirming the evidence already reported by Bonasia et al. [24], which was probably related to the high plant vigour of this landrace. In more detail, CPL accumulated nitrates mostly in the root flesh, while CPT and ‘Presto’ had much lower nitrates in both portions. However, NO_3_ concentration calculated on the intact roots was found to be very low (74.9 mg kg^−1^ fw, on average), showing that carrots grown in the *arenili* accumulate fewer nitrates compared with the carrot varieties grown in other areas [18,34,35], confirming our previous evidence [24]. It can be stated that the carrot genotypes grown in the *arenili* of the SMS area can be included among the “very low” nitrate-accumulating vegetables [36]. Since the nitrate assumption represents a risk for human health [37], the studied genotypes can be considered high-quality carrots.

### 3.3. Simple Sugars

Considering the simple sugars (glucose, fructose, and sucrose) (Table 4), the CPL landrace and the commercial genotype had the highest total and individual concentrations in the peeled root (*p* ≤ 0.001). ‘Presto’ showed a considerable decrease in total sugars concentration in the peels, with values that were the lowest (*p* ≤ 0.001) compared to both landraces. The decrease in the total sugar content in the peel of the commercial genotype substantially involved sucrose (−58%) but also glucose and fructose. For the landraces, a milder reduction in sucrose concentration in the peel was observed (−38% and −19% CPL and CPT, respectively). This suggests that the hybrid ‘Presto’ probably differs from landraces due to a more pronounced radial distribution of storage compounds in the root. The changes in sweetness index (Table 4) were consistent with the free sugars content, with the inner part of CPL and ‘Presto’ showing the highest value, while the peel of commercial genotype having the lowest one (*p* ≤ 0.001).

Irrespectively of the genotype, glucose, fructose, and sucrose concentration appeared greater in the root flesh (Table 4). This result was expected as the inner part of carrot root mainly consists of xylem (core) and phloem (cortex) tissues where sugars are reported to accumulate, especially in the innermost phloem [15]. This evidence may also explain the higher DM detected in the peeled carrots, as DM is correlated with the free sugars content [38].

Since sugars are scarcely present in the periderm [15], the sugar level in the peels detected in this study may be due to the contribution of part of the cortex (outer/middle phloem), which was probably removed by manually peeling the carrots.

Very scarce information is available about the comparison between peeled carrots and waste peripheral tissues in simple sugar concentration. Talcott et al. [13] indicated an unchanged value in carrot puree obtained from root flesh (removing approximately 2 mm of external tissue) or with intact roots, suggesting a very low contribution of peel to the sugars in the puree. In our study, the peel of each genotype provided 7% (’Presto’), 8% (CPL), and 12% (CPT) of the total sugar concentration in intact carrot, as the manually removed peel accounts for 11% (‘Presto’ and CPL, on average) or 15% (CPT) of fresh weight.

In comparison with the total free sugars reported in the previous work on the same genotypes from the same environment [24], generally greater levels (7.8, 6.6, and 7.6 mg 100 g^−1^ fw of the intact root of CPL, CPT, and ‘Presto’, respectively) were observed. These values are comparable with those reported in the national, European, and U.S. food composition databases and several studies (ranging from 4.3 to 13.6 g 100 g^−1^ fw) reviewed in Bonasia et al. [24]. Moreover, the occurrence of sucrose, glucose, and fructose were confirmed, but their relative ratio differed as sucrose was the main sugar in each genotype and root portion (Table 4), while fructose and glucose accounted for 25%, on average, of the total sugar concentration. Overall, these results suggest that in this study, milder stressing conditions linked to specific soil characteristics of the *arenili* seem to occur compared with the previous work [24], where frequent flooding was reported to cause low contents of both sugars and organic acids, along with a faster root maturity, which is underlined by the almost exclusive presence of sucrose.

### 3.4. Carotenoids

It is well known that the consumption of carotenoids is important for human health, as they act as a protector of DNA and other compounds against oxidative damage and contribute to the functionality of the immune system. In particular, β-carotene mainly has pro-vitamin A activity, which is essential for normal organogenesis, immune functions, tissue differentiation, and eyesight. The xanthophyll lutein is also reported to be essential for healthy eyes, and it is involved in cognitive health and in contrasting muscular degeneration during old age [9].

Total carotenoids analysis for the studied genotypes highlighted the occurrence of lutein in both peel and root flesh along with β-carotene, which was predominant (almost 99%) (Table 5), as expected for orange carrot.

As far as β-carotene is concerned, a non-univocal pattern of concentration changes in root portions between genotypes emerged. In the root flesh, the landrace CPL had a greater β-carotene concentration compared with ‘Presto’ and in particular with CPT, while both the landraces had a similar β-carotene concentration in the peel, as CPT showed a much higher value than in root flesh. In ‘Presto’, the level of this carotenoid remained substantially unchanged in root flesh and peel. As a consequence, the contribution of peel to the total β-carotene in intact roots was 30%, 12%, and 13%, respectively, for CPT, CPL, and ’Presto’. Similar behaviour was also observed for the lutein concentration, specifically for CPT, which showed a higher concentration in the peel.

To date, no multiple cultivar comparison has been published on carotenoid distribution in different root tissues. Talcott et al. [13] found a carotenoid content slightly higher in roots with intact periderm, whereas Kenny and O’Beirne [12], studying a different carrot variety, reported a contribution of 28% of the peel to the carotenoid content in intact roots. In “yellow-purple Polignano carrot”, a landrace, β-carotene was found to accumulate more at the cortex level, which included the periderm, than in the root core [20]. In line with our results, these findings may suggest that the accumulation of carotenoids in the peel is genotype dependant.

The β-carotene concentration in unpeeled roots was 28.8, 14.9, and 18.8 mg 100 g^−1^ fw, respectively in CPL, CPT, and ‘Presto’. These values are greater than values for orange carrots reviewed by Arscott and Tanumihardjo [3] or those reported for landraces such as “yellow-purple Polignano carrot” [18,20] and “Tiggiano” carrot [2], and the same orange commercial cultivar (‘Presto’) [18] grown in the Puglia region and other areas worldwide [32,39]. The lutein concentration was in line with values reported by other authors [2,20,32,40] for yellow, orange, and purple carrots.

Despite the variability observed between genotypes, the averaged value (20.9 mg 100 g^−1^ fw) was very similar to that found in Bonasia et al. [24], thus corroborating the evidence that carrots grown in the *arenili* are a high-nutrition product, particularly due to their β-carotene concentration.

### 3.5. Organic Acids (OA)

Except for malic acid [15], no information is available on changes in inorganic acid concentration in different parts of carrot roots. To the best of our knowledge, this is the first study accounting for a comprehensive evaluation of OA distribution in carrot portions also considering a genotype effect.

The concentrations of total OA were higher by 69%, 107%, and 139% in the peel (Table 5) than in the root flesh in CPL, ‘Presto’, and CPT, respectively. The contribution of peel in terms of OA in intact carrots ranged from 19% in CPL and ’Presto’ to a higher 30% in CPT.

In this study, six OA were detected (Table 5), and in agreement with several previous studies [2,24,41], malic acid was the most abundant (92% of total OA) both in the peeled roots and the peripherical tissues. In line with Korolev et al. [15], who found malic acid accumulation in the periderm, our results show a considerably higher concentration of malic acid in the peel, in particular in the CPT landrace, which on the contrary showed the lowest value in the root flesh.

For each of the other detected OA, the generally lower concentration in peeled roots was confirmed with some genotypic exceptions.

A greater level of oxalic acid was observed in the root flesh (*p* < 0.001) of ‘Presto’ and CPT and in the peel (*p* < 0.001) of the commercial genotype and CPL, highlighting an opposite trend between landraces. The oxalic acid occurrence has been indicated by other authors [42] at a slightly greater (3.2 mg kg^−1^ dw) amount than the root flesh of our genotypes (2.3 mg kg^−1^ dw, on average). Nevertheless, Yusuf et al. [43], comparing several carrot varieties reported much higher oxalic concentration values (170–580 mg 100 g fw). It is considered an anti-nutritional compound resulting in the dietary Ca availability reduction and kidney stone disease [44]. Hence, in vegetables, a safe ratio of “oxalic acid/Ca” lower than 2.5 has been established [45]. In the studied carrot varieties, this threshold was never exceeded, highlighting a maximum value of 1.9, which occurred in carrots without peeled CPT and ‘Presto’. A much lower “oxalic acid/Ca” ratio (0.6) was registered in the peeled CPL, as it showed scarce oxalic acid accumulation in the inner part of the root.

Pyruvic acid is not frequently found in carrots; however, it was previously detected by Tsuchida et al. [46], Phan and Hsu [47], and more recently by Šink et al. [41] and Abbey et al. [48]. This study demonstrates a pyruvic acid accumulation in the peripheral tissues, especially of the hybrid Presto, and it confirms the effect of genotype on its accumulation, as suggested by Šink et al. [41]. Since pyruvic acid was the second most abundant organic acid in the peel and it was almost exclusively accumulated in this portion, it emerges that manual peeling, especially in the root flesh of the commercial genotype, may drastically reduce this organic acid, potentially affecting the taste [2].

Ascorbic acid is important for its antioxidant properties, and it represents the most relevant biologically active form of vitamin C. The commercial genotype had the highest ascorbic acid in both root portions, whereas slight differences were detected only in peeled roots between the two landraces. Its concentration in intact carrots was, respectively, 3.0, 3.6, and 4.8 mg 100 g^−1^ fw in CPL, CPT, and ‘Presto’, which is in line with other authors [24,42,49]. However, it is very low, confirming that this vegetable is not a good source of this antioxidant compound [32,41].

The ascorbic acid accumulated in the peel accounts for 23% (CPL and ‘Presto’, on average) and 28% (CPT) of the content in intact roots, and it may be reduced by the manual peeling of carrots. However, considering the total content, the impact of peeling on the ascorbic acid of carrots can be assumed to be negligible. In agreement with this hypothesis, it was found that hand peeling did not change ascorbic acid compared to the intact roots [12].

It is remarkable that in this study, quinic acid was not detected; on the contrary, it occurred in the same varieties obtained in the *arenili* analysed by Bonasia et al. [24]. As quinic acid accumulation is correlatable to cultivation stress conditions (e.g., flooding), it can be argued that not only OA total content but also OA profile may change as affected by exposure to stress factors.

### 3.6. Phenols

Vegetables contain several phenolic compounds that act as antioxidants. Their assumption can result in many beneficial effects on human health by contrasting the occurrence of cardiovascular diseases, cancers, and neurodegenerative diseases [50]. The predominant phenolic compounds in carrots are reported to be phenolic acids [10,50,51], which are simple phenols encompassing hydroxycinnamic acid and its derivatives and hydroxybenzoic acids and its derivatives. Phenolic acids are powerful antioxidants and have been reported to have antibacterial, antiviral, anticarcinogenic, anti-inflammatory and vasodilatory actions [52]. Despite hydroxybenzoic acids having been found in carrots [52], the hydroxycinnamic acids are the main [11] or exclusive phenolic acids occurring in this vegetable [10,24].

**Figure 1:**
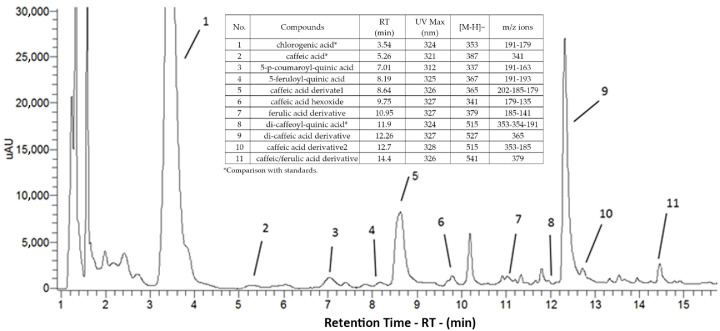
UHPLC chromatogram of peels of ‘Carota a punta lunga’ landrace phenolic compounds.

The exclusive occurrence of hydroxycinnamic acids was confirmed for carrots grown in the *arenili* (Table 6; Figure 1). In terms of qualitative profile, the phenolic compounds were in line with those observed in many studies on carrot reporting chlorogenic acid as prevalent [10,11,20,41], so it substantially affected the total phenolic acid concentration changes observed between varieties in both root portions.

In peeled carrots, irrespective of genotype, the most abundant compounds were chlorogenic acid followed by 5-p-coumaroyl-quinic acid, while all other phenolic compounds were in amounts lower than 1 mg kg^−1^ fw. The root flesh of ‘Presto’ and particularly of the landrace CPT contained the highest level of total phenolic compounds (*p* ≤ 0.05) (specifically, chlorogenic and 5-p-coumaroyl-quinic acids).

Averaged over genotypes, chlorogenic acid in peel was followed by di-caffeic acid derivative, caffeic acid derivative 1, 5-p-coumaroyl-quinic acid, ferulic acid derivative, and caffeic/ferulic acid derivative. All other phenolic acids were in amounts lower than 1 mg kg^−1^ fw. By considering the genotype effect, it emerged that the peel of ‘Presto’ had the lowest concentration (*p* ≤ 0.05) of phenolic acids, while both landraces were 23-fold richer in comparison to the hybrid due to the contribution of chlorogenic acid and di-caffeic acid derivative (*p* ≤ 0.05).

In previous works, a decreasing phenolic acid concentration was found passing from peel to cortex (3.2 times lower) and core (9.5 times lower) [11]. Moreover, hand peeling proved to appreciably reduce the total and individual phenolic acid content in comparison with the intact roots [10,12,13]. However, this latter research did not take into account the effect of genotype on phenolic acid partition in carrot roots.

For the “yellow-purple Polignano carrot” landrace and a commercial genotype grown in the Puglia area, both anthocyanin and phenolic acids accumulation were also registered in the cortex (including the periderm) at double the rate compared to the core portion [20]; however, the comparison between varieties for phenolic acid content is not reported.

Our results confirm a higher concentration of phenolic acids in the peel (Table 6); however, they allow us to highlight that it was true only for the landraces. These latter showed a huge change of phenolic acid concentration between the peel and the root flesh (130.8 vs. 12.9 mg kg^−1^ fw, on average); in contrast, phenolic acid concentrations seem scarcely variable between tissues in the commercial variety. As a consequence, the contributions of the peel of CPL, CPT, and ‘Presto’ were, respectively 82%, 38%, and 5%.

By considering the intact roots, it was pointed out that the richest genotype in phenols was CPT (32.6 mg kg^−1^ fw), as observed in the previous work [24], whereas 19.2 and 12.5 mg kg^−1^ fw of phenols were detected in CPL and ‘Presto’, respectively. However, for each variety in this research, much lower values were observed compared to Bonasia et al. [24], confirming the hypothesis that less stressful growing conditions occurred. In both studies, phenol contents showed a relatively high variability both in landraces and hybrid, suggesting that the biosynthesis of these compounds may be highly affected by the on-field soil variability.

Despite the seasonal variability in phenol content, considerably higher levels of these compounds can be confirmed in carrots cultivated in the *arenili* (216.5 mg kg^−1^ dw, on average). Indeed, lower values have been reported for the orange landrace “Tiggiano” (2.2 mg kg^−1^ dw) [2], an orange commercial variety (80 mg kg^−1^ dw) [20] from the Puglia region, and for other varieties reviewed in Bonasia et al. [24].

### 3.7. Principal Component Analysis and Conclusions

**Figure 2:**
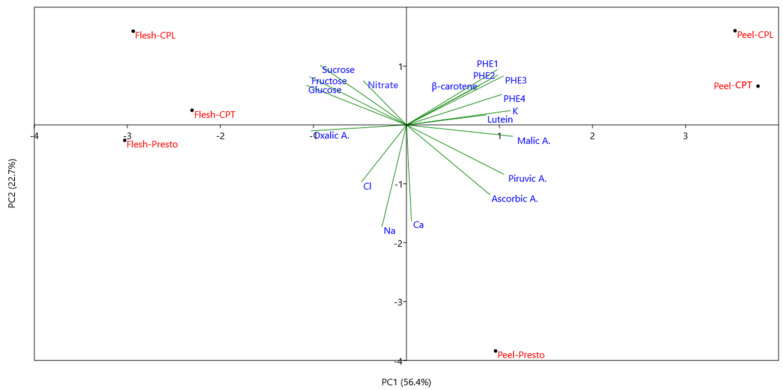
Principal Component Analysis bi-plot (PC1 vs. PC2) reporting the main chemical traits of carrots as affected by peeling and genotypes. The PHEx acronym indicates chlorogenic acid (PHE1), the di-caffeic acid derivative (PHE2), the caffeic acid derivate1 (PHE3), and 5-p-coumaroyl-quinic acid (PHE4). Please see Table 1 for the acronyms of carrot genotypes.

The Principal Component Analysis (PCA), carried out on chemical data, shows that the first two PCs explain approximately 80% of the total variability, attributing 56.1% to PC1 and 23.4% to PC2 (Figure 2).

PC1 discriminates between the peel of the three genotypes, grouped on the right-hand side of the axis (positively correlated to PC1), from the carrot flesh allocated on the left. The peel clustered for the higher concentration of malic, pyruvic, and ascorbic acids, K, the most representative phenolic acids (chlorogenic, di-caffeic derivative, caffeic derivate1, and 5-p-coumaroyl-quinic acid), and carotenoids (β-carotene and lutein). Within this group, PC2 allows the separation of the genotypes with the two landraces, which are positively correlated to PC2 (upper side of PC2), standing out for the accumulation of the phenolic acids and β-carotene, whereas the peel of the commercial genotype ‘Presto’ distinguished for the ascorbic and pyruvic acids as well as Ca concentration, as also emerged from the ANOVA. In addition, it is possible to speculate that among the nutrients, potassium is not correlated with sugars and scarcely correlates with β-carotene, as also reported in Seljåsen et al. [14], whereas it appears strictly correlated to lutein content.

The root deprived of peel clustered on the left-hand side of the PC1 axis for the accumulation of simple sugars, nitrate (mainly in CPL), and oxalic acid, mainly in CPT and ‘Presto’. PC2 separates the two landraces from the commercial genotype also for the root flesh characteristics. However, the differences of local varieties in comparison to ‘Presto’ appear to be at a lower extent than those pointed out for the peel, with the peeled root of ‘Presto’ containing much greater levels of inorganic ions such as Na and Cl. Moreover, the commercial cultivar root flesh shows a lower content of the most important antioxidant compounds and potassium compared to the landraces, especially CPL.

Overall, this evidence highlights that in general, manual peeling may reduce the malic acid and other OAs. Nevertheless, in the landraces, peeling may also result in a larger reduction in phenolic acids, carotenoids, and potassium than in the commercial variety.

In previous studies, by comparing different handling of fresh carrots, hand peeling has resulted in a much higher impact on phenol content than root polishing [10] or on phenols and carotenoids than root abrasion [12]. Nevertheless, no genotype comparison was performed in this latter research.

This study points out that peeling affects the nutritional and antioxidative properties of carrots to a different extent according to genotype. Specifically, for local varieties, the peel removal seems to impact these traits more consistently compared to the advanced cultivar. This information may be useful to support the preference of consumers for landraces by advertising intact root consumption.

It can also be speculated that peeling, by depriving roots of organic acids, could improve the taste and flavour of carrot root flesh, resulting in a higher sweetness perception [2]. Furthermore, the reduction in phenols could result in a lower bitterness [38], mostly for the landraces. However, a sensorial analysis was not performed in this study; therefore, considering that other components may also influence carrot organoleptic characteristics [53], further investigation is needed about these traits and how they are affected by peeling.

## Figures and Tables

**Table 1:** Aerial part characteristics and root bio-morphological features of carrot genotypes.

Genotype ^1^	Leaves		Root					
	Fresh Weight (g)	Number (no.)	Fresh Weight (g)	Length (mm)	Equatorial Diameter (mm)	Dry Mass Concentration		Peel/Total Fresh Weight (%)
						Root Flesh	Peel	
						(g kg^−1^ fw)		
CPL	48.2 (4.9) a ^2^	8.2 (0.5) a	248.8 (21.2) a	231.1 (4.8) a	38.7 (1.2) a	102.8 (0.7) b	92.8 (0.7) b	9.8 (1.0) b
CPT	17.4 (1.9) b	6.5 (0.3) b	110.4 (7.50) b	182.7 (2.5) c	30.3 (0.3) b	93.9 (0.9) c	81.6 (1.5) c	14.6 (1.5) a
Presto	11.7 (1.0) b	5.7 (0.3) b	135.4 (10.0) b	210.8 (6.7) b	31.4 (0.9) b	117.1 (0.6) a	103.3 (0.5) a	12.1 (0.6) b
Significance ^3^	***	***	***	***	***	***	***	***

^1^ CPL, “Carota punta lunga”; CPT, “Carota punta tonda”; “Presto”, hybrid; ^2^ Means (*n* = 15 for leaves; *n* = 6 for roots) (standard error) in columns not sharing the same letters are significantly different according to the LSD test (α = 0.05); ^3^ ***: significant at *p* ≤ 0.001.

**Table 2:** Concentration of cations in the root flesh and peel of carrot genotypes (mg 100 g^−^^1^ fw).

Genotype ^1^	Na	K	Mg	Ca	Total Cations	Na/K
	Root flesh					
CPL	21.2 (0.9) b ^2^	94.6 (3.4) a	2.1 (0.3) b	7.3 (0.5) b	125.2 (4.0) a	0.2 (0.013) b
CPT	18.3 (1.4) b	59.2 (4.4) b	1.2 (0.1) b	7.4 (0.6) b	86.1 (5.9) b	0.3 (0.008) b
Presto	34.5 (2.5) a	40.3 (3.7) c	8.6 (4.5) a	14.6 (4.1) a	98.0 (13.2) b	0.9 (0.093) a
Significance ^3^	***	***	*	*	**	***
	Peel					
CPL	15.4 (0.8) b	178.4 (5.2) a	1.3 (0.1) b	10.6 (0.8) b	205.6 (5.6) a	0.1 (0.006) b
CPT	12.5 (1.3) b	161.4 (10.4) b	1.0 (0.4) b	8.0 (0.7) c	182.8(11.0) b	0.1 (0.010) b
Presto	54.2 (1.4) a	123.6 (4.9) c	7.7 (0.6) a	19.3 (1.1) a	204.9 (5.7) a	0.4 (0.017) a
Significance ^3^	***	***	***	***	**	***

^1^ CPL, “Carota punta lunga”; CPT, “Carota punta tonda”; “Presto”, hybrid; ^2^ Means (*n* = 9) (standard error) in columns not sharing the same letters are significantly different according to the LSD test (α = 0.05); ^3^ *, ** and ***: significant at *p* ≤ 0.05, *p* ≤ 0.01 and *p* ≤ 0.001, respectively.

**Table 3:** Concentration of inorganic anions in the root flesh and peel of carrot genotypes (mg 100 g^−1^ fw).

Genotype ^1^	Cl	NO_3_	PO_4_	SO_4_	Total
	Root flesh				
CPL	42.0 (0.9) b ^2^	15.4 (0.5) a	26.0 (0.8) a	18.8 (0.6) b	102.2 (1.8) a
CPT	44.7 (0.8) a	1.0 (0.5) b	6.9 (0.6) c	10.1 (0.3) c	62.8 (1.3) c
Presto	42.6 (1.0) b	2.0 (0.6) b	19.0 (1.7) b	21.0 (0.3) a	84.6 (2.2) b
Significance ^3^	*	***	***	***	***
	Peel				
CPL	29.1 (0.8) b	4.6 (0.3) a	23.9 (0.6) a	17.9 (0.4) b	75.5 (0.8) c
CPT	44.7 (0.9) a	0.0 (0.0) c	25.6 (1.1) a	16.4 (0.4) c	86.6 (1.7) b
Presto	45.6 (1.2) a	2.3 (0.6) b	23.1 (0.7) a	20.4 (0.4) a	91.4 (2.0) a
Significance ^3^	***	***	ns	***	***

^1^ CPL, “Carota punta lunga”; CPT, “Carota punta tonda”; “Presto”, hybrid; ^2^ Means (*n* = 9) (standard error) in columns not sharing the same letters are significantly different according to the LSD test (α = 0.05); ^3^ ns, * and ***: not significant and significant at *p* ≤ 0.05, *p* ≤ 0.001, respectively.

**Table 4:** Concentration of simple carbohydrates and carotenoids in the root flesh and peel of carrot genotypes.

Genotype ^1^	Glucose	Fructose	Sucrose	Total Sugars	Sucrose/ Total Sugars	Sweetness Index	Lutein	ß-Carotene
	(g 100 g^−1^ fw)						(µg 100 g^−1^ fw)	
	Root flesh							
CPL	2.1 (0.06) a ^2^	2.1 (0.06) a	3.9 (0.16) a	8.1 (0.21) a	0.48 (0.01) ab	12.1 (0.31) a	307 (46) a	28,430 (1304) a
CPT	1.8 (0.04) b	1.8 (0.03) b	3.1 (0.05) b	6.8 (0.09) b	0.46 (0.01) b	10.2 (0.13) b	122 (14) b	12,336 (471) c
Presto	2.1 (0.03) a	2.0 (0.02) a	4.0 (0.05) a	8.0 (0.08) a	0.49 (0.01) a	12.0 (0.13) a	270 (73) a	18,368 (2348) b
Significance ^3^	***	***	***	***	**	***	*	***
	Peel							
CPL	1.5 (0.05) a	1.5 (0.03) a	2.4 (0.07) a	5.4 (0.10) a	0.45 (0.01) a	8.2 (0.15) a	349 (24) b	31,849 (1060) a
CPT	1.4 (0.07) b	1.5 (0.04) a	2.5 (0.05) a	5.3 (0.13) a	0.46 (0.01) a	8.1 (0.19) a	433 (32) a	29,629 (1689) a
Presto	1.4 (0.05) b	1.4 (0.04) a	1.7 (0.07) b	4.6 (0.14) b	0.37 (0.01) b	7.0 (0.21) b	312 (38) b	22,682 (2150) b
Significance ^3^	***	ns	***	***	***	***	**	***

^1^ CPL, “Carota punta lunga”; CPT, “Carota punta tonda”; “Presto”, hybrid; ^2^ Means (*n* = 9) (standard error) in columns not sharing the same letters are significantly different according to the LSD test (α = 0.05); ^3^ ns, *, ** and ***: not significant, significant at *p* ≤ 0.05, *p* ≤ 0.01 and *p* ≤ 0.001, respectively.

**Table 5:** Concentration of organic acids in the root flesh and peel of carrot genotypes (mg 100 g^−1^ fw).

Genotype ^1^	Oxalic Acid	Pyruvic Acid	Ascorbic Acid	Citric Acid	Malic Acid	Succinic Acid	Total	Oxalic Acid/Ca
				Root flesh				
CPL	3.8 (0.1) c ^2^	1.4 (0.1) a	2.6 (0.1) c	1.9 (0.3) a	311.4 (7.5) a	19.9 (1.3) a	341.0 (8.5) a	0.6 (0.1) b
CPT	12.6 (1.3) b	0.9 (0.1) b	3.0 (0.1) b	0.4 (0.1) a	274.3 (20.8) b	6.0 (0.2) b	297.3 (13.8) b	1.9 (0.2) a
Presto	16.4 (0.3) a	0.4 (0.0) c	4.1 (0.1) a	1.4 (0.5) a	246.4 (11.2) c	1.9 (0.2) c	270.7(11.0) c	1.9 (0.2) a
Significance ^3^	***	***	***	***	***	***	***	***
				Peel				
CPL	10.4 (0.5) b	15.3 (1.5) b	6.8 (0.3) b	1.1 (0.1) a	531.6 (8.0) b	9.9 (0.2) b	575.1 (9.2) b	1.1 (0.1) a
CPT	3.5 (0.3) c	18.2 (1.2) ab	6.7 (0.2) b	0.0 (0.0) b	672.6 (25.1) a	8.8 (0.5) b	709.7 (25.0) a	0.5 (0.1) b
Presto	12.4 (0.2) a	21.5 (1.4) a	9.3 (0.2) a	0.0 (0.0) b	513.4 (9.8) b	4.4 (0.1) c	560.9 (10.0) b	0.7 (0.1) b
Significance ^3^	***	*	***	***	***	***	***	***

^1^ CPL, “Carota punta lunga”; CPT, “Carota punta tonda”; “Presto”, hybrid; ^2^ Means (*n* = 9) (standard error) in columns not sharing the same letters are significantly different according to the LSD test (α = 0.05); ^3^ *, and ***: significant at *p* ≤ 0.05 and *p* ≤ 0.001, respectively.

**Table 6:** Concentration of phenolic compounds in the root flesh and peel of carrot genotypes (mg kg^−1^ fw).

Genotype ^1^	Chlorogenic Acid ^4^	Caffeic Acid ^4^	5-p-Coumaroyl-quinic Acid	5-Feruloyl-quinic Acid	Caffeic Acid Derivate 1	Caffeic Acid-o-hexoside	Ferulic Acid Derivative	Di-caffeoyl-quinic Acid ^4^	Di-caffeic Acid Derivative	Caffeic Acid Derivative 2	Caffeic/ferulic Acid Derivative	Sum of Un-identified Phenols	Total Phenols
	Root flesh												
CPL	1.7 (1.1) b ^2^	0.002 (0.0003) b	0.09 (0.03) b	0.10 (0.04) b	0.28 (0.22) a	0.07 (0.02) a	0.03 (0.02) a	0.002 (0.001) a	0.58 (0.46) a	0.01 (0.001) a	0.03 (0.02) a	0.33 (0.12) a	3.2 (1.9) b
CPT	15.9 (1.3) a	0.009 (0.0003) a	3.10 (1.1) a	0.84 (0.25) a	0.90 (0.45) a	0.07 (0.02) a	0.11 (0.05) a	0.007 (0.003) a	1.34 (1.06) a	0.02 (0.003) a	0.06 (0.03) a	0.21 (0.12) a	22.6 (2.6) a
Presto	9.1 (4.8) ab	0.014 (0.002) a	1.59 (0.50) ab	0.71 (0.26) ab	0.63 (0.35) a	0.06 (0.01) a	0.10 (0.03) a	0.072 (0.011) a	0.73 (0.64) a	0.05 (0.01) a	0.05 (0.02) a	0.30 (0.13) a	13.4 (6.3) ab
Significance ^3^	*	**	*	*	ns	ns	ns	ns	ns	ns	ns	ns	*
	Peel												
CPL	54.5 (8.5) a	0.005 (0.002) a	5.8 (1.0) a	2.3 (0.5) a	23.6 (5.1) a	0.25 (0.03) a	2.3 (0.3) a	0.253 (0.107) a	55.4 (13.1) a	1.1 (0.6) a	2.7 (0.7) a	0.30 (0.05) a	149.5 (28.4) a
CPT	51.1 (7.2) a	0.003 (0.0003) a	7.8 (0.9) a	3.2 (1.3) a	17.1 (5.1) ab	0.31 (0.05) a	2.2 (1.3) a	0.288 (0.114) a	28.5 (9.0) ab	0.5 (0.2) a	2.0 (1.2) a	0.18 (0.04) a	113.2 (25.5) a
Presto	0.7 (0.3) b	0.004 (0.0003) a	1.8 (0.2) b	0.4 (0.09) a	0.9 (0.4) b	0.01 (0.001) b	0.1 (0.05) a	0.002 (0.001) a	1.4 (0.7) b	0.1 (0.02) a	0.08 (0.03) a	0.25 (0.03) a	5.7 (1.4) b
Significance	*	ns	*	ns	*	***	ns	ns	*	ns	ns	ns	*

^1^ CPL, “Carota punta lunga”; CPT, “Carota punta tonda”; “Presto”, hybrid; ^2^ Means (*n* = 9) (standard error) in columns not sharing the same letters are significantly different according to the LSD test (α = 0.05); ^3^ ns, *, **, ***: not significant, and significant at *p* ≤ 0.05, *p* ≤ 0.01, *p* ≤ 0.001 respectively; ^4^ Comparison with standards.

## Data Availability

The data presented in this study are available on request from the corresponding author. The data are not publicly available due to privacy restrictions.

## Supplementary Materials

The following supporting information can be downloaded at: https://www.mdpi.com/article/10.3390/foods11010045/s1, Figure S1: Cultivation area of the ‘Carota a punta lunga’, ‘Carota a punta tonda’ and carrot hybrid Presto. The most typical area is along the cost between Margherita di Savoia and Zapponeta; Figure S2: Intact roots of ‘Carota a punta lunga’ (a), ‘Carota a punta tonda’ (b), carrot hybrid Presto (c), and their longitudinal sections (d), (e), (f).
