# Supplementary figures and images for: Peeling Affects the Nutritional Properties of Carrot Genotypes
Source: Foods. 2021 Dec 24;11(1):45. doi: 10.3390/foods11010045 (PMC8750513; doi:10.3390/foods11010045)

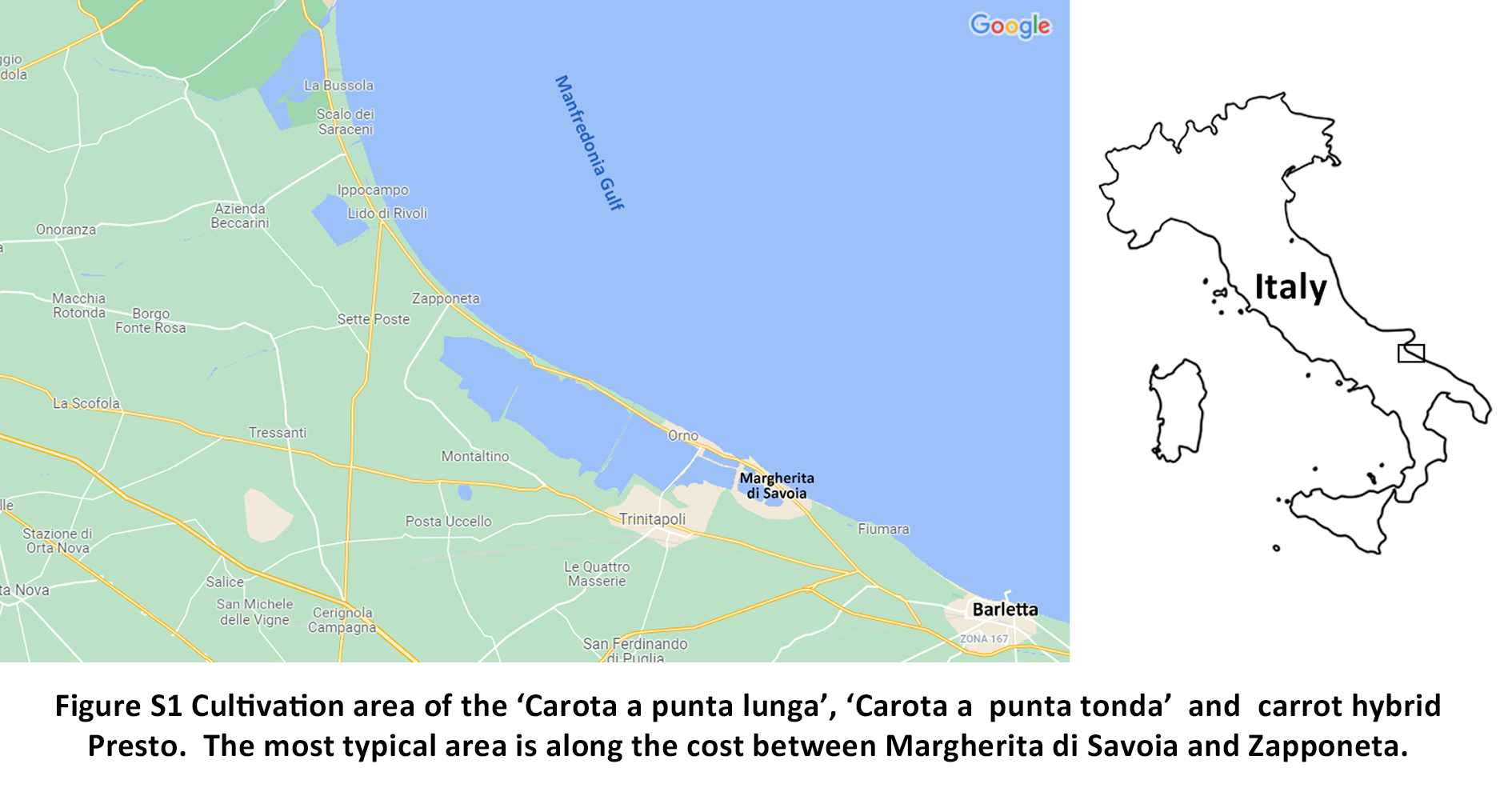

Supplement: Supplementary file 1 [file foods-11-00045-s001.zip › Figure S1.tif]

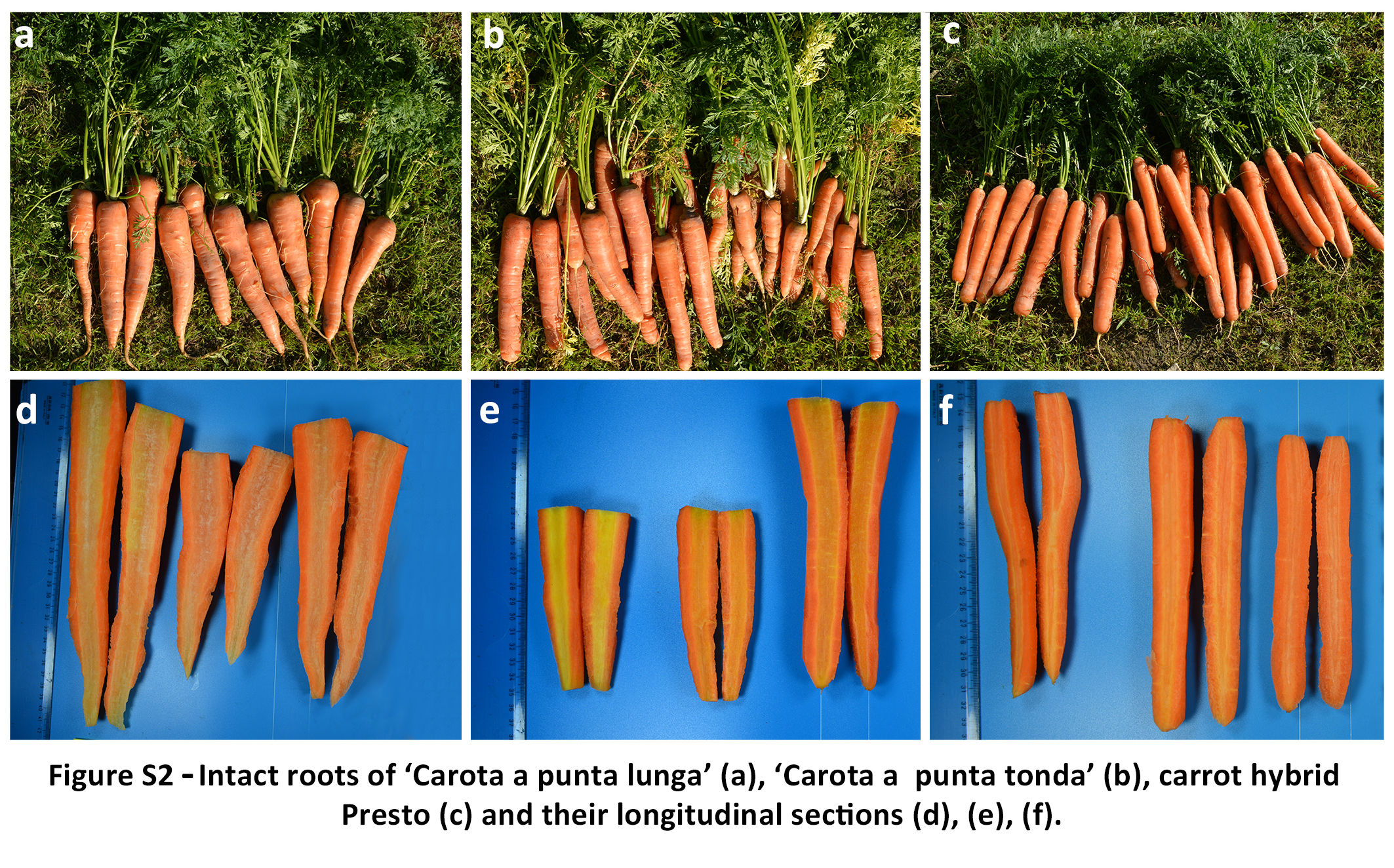

Supplement: Supplementary file 1 [file foods-11-00045-s001.zip › Figure S2.jpg]
